# Cardiac troponins: from myocardial infarction to chronic disease

**DOI:** 10.1093/cvr/cvx183

**Published:** 2017-09-14

**Authors:** Kyung Chan Park, David C Gaze, Paul O Collinson, Michael S Marber

**Affiliations:** 1 1 BHF Centre of Research Excellence, The Rayne Institute, Cardiovascular Division, King’s College London, London, UK;; 2 2 Burdon Sanderson Cardiac Science Centre, Department of Physiology, Anatomy and Genetics, University of Oxford, Oxford, UK;; 3 3 Clinical Blood Sciences and Cardiology, St George’s University Hospitals NHS Trust and St George’s University of London, London, UK;; 4 4 Department of Biomedical Science, University of Westminster, London, UK

**Keywords:** Cardiac troponin, Chronic disease, Release mechanism, Biomarker, Prognosis

## Abstract

Elucidation of the physiologically distinct subunits of troponin in 1973 greatly facilitated our understanding of cardiac contraction. Although troponins are expressed in both skeletal and cardiac muscle, there are isoforms of troponin I/T expressed selectively in the heart. By exploiting cardiac-restricted epitopes within these proteins, one of the most successful diagnostic tests to date has been developed: cardiac troponin (cTn) assays. For the past decade, cTn has been regarded as the gold-standard marker for acute myocardial necrosis: the pathological hallmark of acute myocardial infarction (AMI). Whilst cTn is the cornerstone for ruling-out AMI in patients presenting with a suspected acute coronary syndrome (ACS), elevated cTn is frequently observed in those without clinical signs indicative of AMI, often reflecting myocardial injury of ‘unknown origin’. cTn is commonly elevated in acute non-ACS conditions, as well as in chronic diseases. It is unclear why these elevations occur; yet they cannot be ignored as cTn levels in chronically unwell patients are directly correlated to prognosis. Paradoxically, improvements in assay sensitivity have meant more differential diagnoses have to be considered due to decreased specificity, since cTn is now more easily detected in these non-ACS conditions. It is important to be aware cTn is highly specific for myocardial injury, which could be attributable to a myriad of underlying causes, emphasizing the notion that cTn is an organ-specific, not disease-specific biomarker. Furthermore, the ability to detect increased cTn using high-sensitivity assays following extreme exercise is disconcerting. It has been suggested troponin release can occur without cardiomyocyte necrosis, contradicting conventional dogma, emphasizing a need to understand the mechanisms of such release. This review discusses basic troponin biology, the physiology behind its detection in serum, its use in the diagnosis of AMI, and some key concepts and experimental evidence as to why cTn can be elevated in chronic diseases.

## 1. Introduction

**Figure 1:**
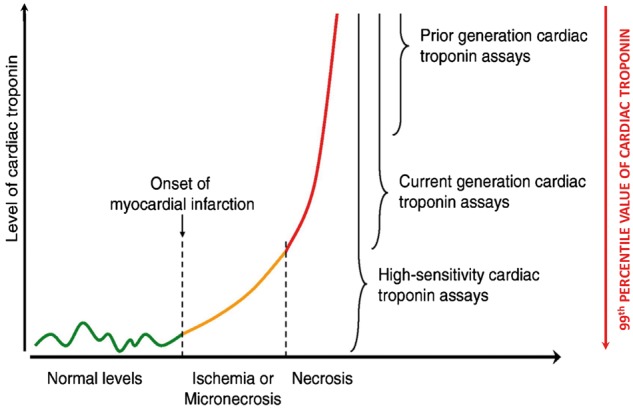
Illustration of the detection ranges of different cardiac troponin (cTn) assays. cTn can be detected in healthy individuals using high-sensitivity assays, possibly due to physiological turnover of cardiac myocytes (green line). Subsequent to an acute myocardial infarction (AMI), there is a slight rise in cTn which may reflect either ischaemia-induced release of the ‘early-release pool’ or micronecrosis (orange line). After ∼2–6 h there is a precipitous rise in cTn reflecting extensive myocardial necrosis and degradation of myofibrillar cTn (red line). With the evolution of assay technology, the 99th percentile value of cTn which serves as a cut-off value for the diagnosis of AMI has been accordingly reduced. Edited from Hochholzer *et al.*^103^ with permission.

Intensive investigation into the mechanisms of striated muscle contraction during the late 50 s and early 60 s led to evidence of a protein that resembled tropomyosin and regulated the calcium sensitivity of the actomyosin contractile apparatus. This finding subsequently led to the discovery of troponin by Ebashi and Kodama in 1965. Elucidation of the physiologically distinct subunits of troponin by Greaser and Gergely^1^ in 1973 has facilitated a quantum-leap in our understanding of the molecular physiology underpinning cardiac contraction. Consequent to their findings, one of the most successful diagnostic investigations to date has been developed: the cardiac troponin (cTn) assays. Whilst troponin is found in all forms of striated muscle, troponin in the heart is distinguished by regions of different amino acid sequences. Identifying the subtle dissimilarities between cardiac and skeletal troponin enabled the raising of antibodies against specific epitopes. These antibodies were exploited to develop myocardial-specific assays. cTn assays have been regarded for the past decade as the gold-standard biomarker for detecting acute myocardial necrosis, the pathological hallmark of acute myocardial infarction (AMI).^2^ It is current routine practice for cTn assays to be run on any patient presenting with a suspected acute coronary syndrome (ACS) to rule-in or rule-out an AMI.^3^ However, compared to when the assays were first developed, sensitivity and analytical performance have improved to such an extent that cTn can be detected in the healthy population (*Figure *1).

Over 50% of patients presenting with chest pain have cTn levels elevated above the population-defined 99th percentile.^4^ Yet, in the absence of any confirmatory clinical signs or ancillary tests, such troponin elevation does not necessarily indicate an AMI. Troponin elevation without AMI often reflects myocardial injury of unknown origin, leaving us with the notion that the cTn assay is organ-specific, not disease-specific. Thus, with the progression of time and assay technology, a multiplicity of confounding factors now have to be considered when interpreting cTn results. Additionally, clinical studies have demonstrated that cTn can be elevated in numerous chronic conditions.

In recent years, the literature suggests cTn can be released with reversible cell injury in the absence of necrosis or cell death. This has been prompted (and reinforced by), observations of increased cTn in clinical situations whereby there is no obvious coronary syndrome, such as extreme exercise. It is also unclear why cTn elevations are seen in chronic diseases in the absence of ACS: yet such cTn elevations are strongly predictive of survival. This review addresses the biology of troponin, the physiology behind its detection in serum, its clinical utilization as a biomarker of AMI and myocardial injury, as well as the experimental evidence behind cTn elevation in several chronic conditions.

## 2. Troponins

### 2.1 Background

The sarcomere is the fundamental contractile unit of the heart comprised of thick-filaments (myosin) and thin-filaments. The thin-filament is a helix of two filamentous-actins (F-actin), which themselves are polymers of globular-actin subunits (G-actin). The groove of the F-actin helix contains tropomyosin to which troponin is attached (the tropomyosin-troponin complex). Myofibrillar contraction is activated by depolarization and then modulated by the interplay of Ca^2+^ with specific regulatory sites on the contractile apparatus of striated muscle.^5^ This regulatory site is the troponin complex, a tadpole-shaped heterotrimer immobilized on the thin-filament, which acts in an allosteric manner to regulate the Ca^2+^-dependent interaction of actin and myosin filaments.^6^

Troponin was thought to be a single homogenous protein until the late 60 s when it was fractionated into two distinct proteins by Hartshorne and Mueller.^7^ Their findings raised the possibility of troponin being a complex of multiple subunits, leading to the landmark study by Greaser and Gergely^1^ in 1973 which introduced the troponin subunit nomenclature still used today. Greaser and Gergely^8^ purified troponin from rabbit skeletal muscle to reveal four major protein fractions using SDS-PAGE. Reconstitution experiments were performed to find fractions 2, 3, and 4 being constitutively required for normal troponin activity. The devised nomenclature was based on the functional properties exhibited by these fractions. Fraction 2 (∼24 kDa) inhibited activity of the Mg^2+^-dependent actomyosin ATPase in the absence of Ca^2+^, thus named *‘I’* for *inhibitory* (TnI). TnI modulates the response to intracellular Ca^2+^ by preventing actin-myosin interactions and cross-bridge formation in the absence of Ca^2+^. Fraction 3 (∼37 kDa) bound to tropomyosin thus named *’T’* for *tropomyosin* (TnT). TnT serves as the mechanical link between tropomyosin (on the thin-filament) and the troponin complex. Fraction 4 (∼20 kDa) was the Ca^2+^-binding subunit and was named *‘C’* for *calcium* (TnC). TnC regulates activation of the thin-filament.^9^^,^^10^

### 2.2 Cardiac-specific isoforms of troponin

To be clinically useful, any biomarker intended for the detection of pathological insults to the heart needs to be highly specific and sensitive. Since both skeletal and cardiac muscle contract via a troponin-dependent mechanism, the question arises as to how to differentiate myocardial injury from skeletal muscle injury. The answer lies in the fact that there are multiple isoforms of each troponin subunit which are encoded by distinct genes, some of which are expressed selectively in cardiac muscle. Whilst TnI and TnT have distinct cardiac and skeletal isoforms, they share a common isoform of TnC: the slow-twitch skeletal muscle isoform (ssTnC) (*Table *1). Thus in the healthy, fully developed heart, cardiac TnI and cardiac TnT (cTnI and cTnT) and c/ssTnC are expressed in combination. There is developmentally regulated expression of certain TnI and TnT isoforms which are downregulated postnatally (commonly referred to as ‘foetal isoforms’).^11^ These foetal isoforms can be re-expressed postnatally under certain pathological circumstances.

**Table 1:** Isoforms of the troponin subunits with their respective genes and expression sites

Troponin I (TnI)		Troponin T (TnT)		Troponin C (TnC)	
Isoform *(Gene)*	Expression site	Isoform *(Gene)*	Expression site	Isoform *(Gene)*	Expression site
**Fast-skeletal** **‘fsTnI’** **(TNNI2)**	Fast-twitch skeletal muscle	**Fast-skeletal** **‘fsTnT’** ***(TNNT3)***	Fast-twitch skeletal muscle	**Fast-skeletal** **‘fsTnC’** ***(TNNC2)***	Fast-twitch skeletal muscle
**Slow-skeletal** **‘ssTnI’** ***(TNNI1)***	Slow-twitch skeletal muscle	**Slow-skeletal** **‘ssTnT’** ***(TNNT1)***	Slow-twitch skeletal muscle	**Slow-skeletal** **‘ssTnC’ or ‘cTnC’** ***(TNNC1)***	Slow-twitch skeletal muscle and cardiac muscle
**Cardiac** **‘cTnI’** ***(TNNI3)***	Cardiac muscle	**Cardiac** **‘cTnT’** ***(TNNT2)***	Cardiac muscle		

The genes specified above have been described on the basis of molecular cloning in humans (OMIM®, 1966–2015). They are expressed in a tissue-specific manner. There is no unique cardiac isoform of TnC and ssTnC is found in both cardiac and skeletal muscle.^11^ For clarity, the literature often refers to ssTnC as cTnC. They are, however, analogous.

The ontology of cTnI is clear. During embryonic and foetal development, ssTnI is expressed exclusively in the heart in lieu of cTnI.^12^ The expression of this foetal isoform is downregulated and switched to cTnI at ∼8–9 months postnatally.^13^ The case for cTnT is more complex. cTnT is unique in that *TNNT2* generates multiple alternatively spliced transcripts encoding different isoforms. Four distinct cTnT isoforms are generated via alternative splicing (designated cTnT_1-4_ numbered in order of decreasing molecular size), where cTnT_3_ is the dominant isoform in the normal adult heart.^14^ In addition to other proteins of the contractile apparatus like myosin,^15^ reversion of troponin to foetal isoforms can occur during disease states such as chronic heart failure. Reversion of cTnI does not occur.^13^ cTnT_2_ is expressed to a significantly greater extent compared to cTnT_3_ in failing hearts.^14^ Since the cTnT assay detects all cTnT isoforms, this has no impact on its clinical performance in heart failure patients.^16^ However, it is worth noting that diseased skeletal muscle has been demonstrated to re-express foetal TnT which is detected by cTnT assays, leading to false-positives.^17^

Normal cardiac function relies on the expression of all three troponin subunits. This is exemplified by *in vivo* murine knockout models. cTnT-knockout causes sarcomere disassembly and early embryonic lethality.^18^ Remarkably, knockout of cTnI in murine embryos has no effect on health before postnatal day 15, attributable to ssTnI compensating for the lack of cTnI.^19^ However, the mice died on day-18 of acute heart failure secondary to TnI deficiency as ssTnI expression was downregulated. There are no reports in the literature of cTnC-knockout studies (presumably it would be lethal).

### 2.3 cTn is special

Since each troponin isoform is encoded by a separate gene, what makes cTn special is fundamentally down to its protein structure. The amino acid (AA) sequences for cTnI and cTnT were first identified in rabbits by Grand *et al.*^20^ and Pearlstone *et al.*^21^ respectively. Identification of the AA sequences for human cTnI and cTnT followed and were first reported by Vallins *et al.*^22^ and Townsend *et al.*^23^ respectively. It was subsequently identified that cTnI and cTnT sequences differed from their skeletal counterparts. For example, cTnI contains 210 AA residues, 31 of which could be utilized for assay development since they form an N-terminus extension not found in skeletal troponin.^22^ We performed a protein alignment using the Basic Local Alignment Search Tool (BLAST), to show that human cTnI and ssTnI were 63% identical and 77% similar, whilst cTnI and fsTnI are 57% identical and 76% similar (‘similar’ denotes AAs that are chemically similar but not identical, i.e. conserved substitutions) (NCBI accession numbers: *TNNI1*, J04760; *TNNI2*, L21715; *TNNI3*, X54163). Since the cTnI assay uses highly specific antibodies, the magnitude of the absolute difference between skeletal and cardiac TnI is not crucial– provided divergent epitopes are chosen carefully. This is evident from the fact that both cTnI and cTnT assays currently in clinical use are highly cardiac selective.

## 3. History of defining MI and cardiac biomarkers

In the simplest terms, ‘myocardial infarction’ means death (necrosis) of the myocardium due to inadequate oxygen supply. In the clinical context however, ‘defining’ myocardial infarction is far more complex due to the number of aspects by which myocardial necrosis can be assessed: histopathologic, biochemical markers, electrocardiographic changes and imaging.^24^ The development of cardiac biomarkers began in the 50 s (see Collinson *et al.*^25^ for review). During the 60 s–70 s, creatine kinase (CK) was shown to have clinical value in ruling-in/out an AMI. In tune with this, a definition of AMI was presented in 1979 by the World Health Organization,^26^ where the clinical diagnosis of an AMI was based on a combination of indices: clinical symptoms (e.g. chest pain), ECG abnormalities (e.g. pathological Q waves), and serial cardiac enzymes (e.g. MB isoenzyme of CK). However, the advent of more sensitive and myocardial-selective biomarkers, combined with improved imaging techniques, meant that very small amounts of myocardial injury and necrosis could be detected–lesions that would not have been detected when technology was less developed, necessitating redefinitions of MI.

Assays for cTnI were first described by Cummins *et al.*^27^ and subsequently for cTnT by Katus *et al.*^28^ In the clinical studies that followed, meta-analyses subsequently demonstrated that cTn was better at predicting future major adverse cardiac events than CK-MB.^25^ Recognizing the diagnostic superiority of cTn over traditional cardiac enzyme assays, the National Academy of Clinical Biochemistry (NACB) subsequently published in 1999 a consensus guideline for the clinical use of cTn assays.^29^ A new definition of MI was presented by the Global MI Task Force in 2000 incorporating the NACB report from 1998, stating that either cTn, or if not available, CK-MB should be used in diagnosing an AMI.^24^

In 2007, the troponin standard was adopted and the use of CK-MB in the diagnosis of AMI was no longer recommended.^30^ Currently, the Third Global MI Task Force defines an AMI as when there is evidence of myocardial necrosis, in a clinical setting consistent with myocardial ischaemia.^2^ In keeping with the previous definitions of MI, such evidence incorporates clinical symptoms of ischaemia, ECG abnormalities and imaging evidence. However, in contrast to the preceding definition, the third definition now explicitly states that detection of a rise and/or fall (i.e. a temporal or kinetic change) of cTn is paramount in making a diagnosis of AMI. The reason why the ‘rise and/or fall’ of cTn is emphasized is attributable to the release kinetics of cTn (Section 4.3).

## 4. Detection of cTn in serum

### 4.1 The cTn assay

The differences in AA sequences permitted development of quantitative assays for cTnI/T. Most cTn assays are non-competitive enzyme-linked immunosorbent assays (ELISA) based on the sandwich principle, utilizing the high specificity and affinity of antibodies.^31^ The assay is based on a capture antibody which binds to a specific epitope of cTn, and a detection antibody which binds to a separate epitope. The epitopes are often closely spaced to prevent a proteolytic cleavage event diminishing sensitivity. Further, the most stable regions of cTn are selected as epitopes; regions that are not susceptible to cleavage or post-translational modifications, e.g. phosphorylation.^16^ The detection antibody is linked to a signal-generating system to enable quantification. Signal amplification is achieved by using an enzyme which can cleave multiple molecules of a substrate over a given time-interval, or by using other detection methodologies such as gold microparticles^32^ and ruthenium.^33^

### 4.2 Multiple circulating forms of cTn

**Figure 2:**
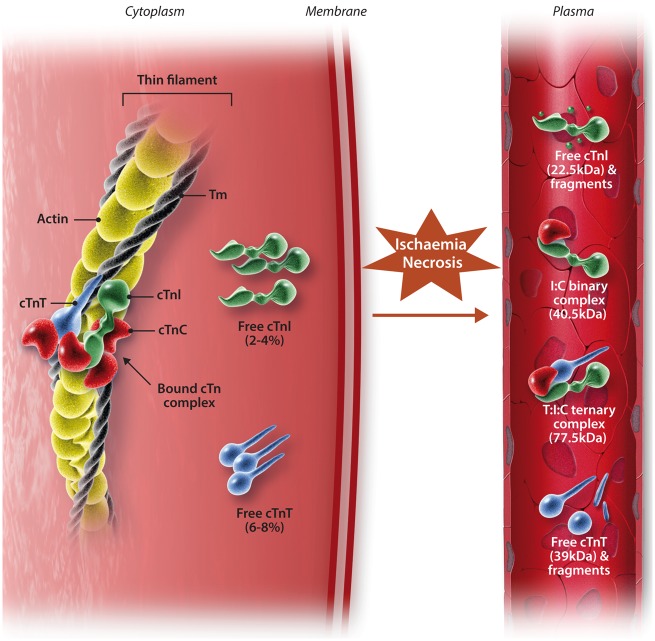
*** ***Structure of the cardiac troponin-tropomyosin complex and the forms of troponin released following myocardial necrosis. Whilst most cardiac troponin (cTn) is bound to the myofibril, there is different subcellular localization of some cTn. ∼2–4% and ∼6–8% of cTnI and cTnT respectively exist either unbound in the cytosol, or loosely bound to the sarcomere. Following myocardial ischaemia, the ensuing necrosis of cardiac myocytes results in different forms of cTn being detectable in serum. After ischaemic insult, the free forms of cTnI and cTnT from the cytosol are soon detected in serum prior to detection of the complexed forms. There are several complexed forms that exist: non-covalent ternary complexed cTnT-I-C (T:I:C complex) and binary complexed cTnI-T (I:T complex). Edited from Gaze and Collinson^34^ with permission.

After the onset of myocardial ischaemia, cardiac myocyte death can occur within 15 min, with histological evidence of necrosis appearing within 4–6 h.^24^ cTn is released from the myocardium a few hours following a period of ischaemia and is detectable in the venous circulation once the interstitial fluid from the infarct zone has been cleared by the cardiac lymphatics.^34^ cTnI/T are not only released in free-forms but also as non-covalent ternary and binary complexes (*Figure *2). Evidence from clinical studies have shown that following AMI, cTnT primarily appears in blood as a mixture of free-forms and the T:I:C ternary complex, whilst cTnI appears predominantly as the I:C binary complex.^35^ In addition, all forms of troponin are open to redox modifications and can exist as oxidized and reduced forms.^35^ Though it is not completely clear exactly which form of cTn is being detected during routine clinical practice, current assays detect these different forms on a near-equimolar basis, so redox changes are unlikely to affect clinical sensitivity.^16^

### 4.3 Subcellular localization and release kinetics

Following an AMI, there is typically an initial peak of serum cTn followed by a sustained elevation, for up to 14 days after symptom-onset (depending on the infarct size).^36^ This observation was made during early clinical studies investigating the performance of cTn assays and at the time raised some questions: (i) why are there differences in release profiles between reperfusion, and non-reperfusion, following AMI; (ii) are there different cellular distributions of cTn since it has a biphasic release profile.

Myocardial reperfusion is the restoration of coronary blood flow following an AMI via thrombolytic therapy, percutaneous coronary intervention, or spontaneous thrombolysis. Reperfusion therapy is established as the most effective strategy for reducing infarct size and improving prognosis.^37^^,^^38^ An early clinical study by Katus *et al.*^39^ showed that reperfusion following AMI results in both cTnT and CK-MB being detected earlier and at greater magnitudes. Furthermore, the elevation of cTnT is biphasic with reperfusion, but monophasic without. Generally, the rate at which any protein is liberated depends upon its intracellular location, molecular weight, and the local blood- and lymphatic-flow. Since reperfusion restores blood flow to the infarct zone, one of the explanations for the earlier detection of cTn in reperfused patients is clearance/washout of the infarct zone. However, since Katus *et al.*^39^ observed the concentration of cTnT peaking at the same time as CK-MB (localized in the cytosol), and since the release profile of cTnT was biphasic, these observations cannot be accounted for by infarct-zone clearance/washout alone.

Through studies employing human myocardium^40^ and a rat Langendorff model,^41^ it was demonstrated that in fact, not all troponin may be bound to the myofilament, which may account for the biphasic release profile. This is demonstrated in a study by Remppis *et al.*^41^ whereby male Wistar rat hearts were homogenized and centrifuged. The derived supernatant was used to measure the soluble cytosolic concentration of cTnT, whilst the resulting pellet was used to measure the myofibrillar fraction of cTnT. Bleier *et al.*^40^ adopted a similar method but using fresh human right atrial appendages, freshly excised from patients with normal chamber pressures, to eliminate any bias.

It is thought that the serum concentration profile of cTn in reperfused patients following an AMI is attributable to the findings from these early studies, with release from a ‘cytosolic pool’ contributing to an initial peak (*Figure *2). Degradation of the contractile apparatus (‘structural pool’) contributes the sustained elevation, which also reflects infarct size.^16^^,^^42^ This is substantiated by findings of cTn in the free-form (‘cytosolic pool’) being detected shortly after the ischaemic insult, prior to detection of the binary- and ternary-complexed forms of cTn.^34^ It should be noted that whilst the release profile of cTnT is well-established as being biphasic, for cTnI, it is recognized as being monophasic, lacking an initial early peak.^4^ This has been suggested to be due to the cytosolic pool of cTnI being smaller, although in practice there appears to be a similar early rise of cTnI of a smaller magnitude.^9^

As an aside, the common usage of the term ‘cytosolic pool’ has been challenged by several authors.^16^^,^^43^ cTnI/T has poor solubility in the hydrophilic cytoplasm, thus it has been inferred that they may simply be more loosely bound to the myofibril, as opposed to it being completely isolated in the cytoplasm. Therefore, it is suggested the term ‘early-release pool’ may be a more accurate term to describe the initial peak in cTn.

### 4.4 Biochemical mechanisms of troponin cleavage

Troponin is released from the myofibril due to proteolytic degradation in the myocardium, both as intact proteins and as degradation products.^16^ So far, three myocardial enzymes have been implicated: (i) calpain 1, a Ca^2+^-dependent cysteine protease^44^; (ii) caspase, a cysteine protease involved in mediating apoptosis; (iii) matrix metalloproteinase-2, a zinc-dependent endopeptidase.^45^ These enzymes are also present in blood and the complexes of cTnI (T:I:C and I:C complex) are susceptible to degradation in the circulation.^16^ The N- and C-terminal regions of cTnI are the most susceptible to proteolysis. The central region of cTnI (residues 30-110) is the Ca^2+^-dependent TnC binding domain and is the most stable.^9^ As such, this is the region currently targeted by most cTnI assays.^34^

## 5. Clinical uses of cTn

### 5.1 ACS

Acute coronary syndrome (ACS) is an umbrella term for ST-segment-elevation MI (STEMI), non-ST-segment-elevation MI (NSTEMI) and unstable angina, which share presenting symptoms but differ in underlying pathology.^3^ Myocardial ischaemia is the first step in developing an MI and occurs as a result of oxygen supply-demand mismatch^2^ or reduced coronary flow.^46^ The pathophysiology underlying ACS is atherosclerotic plaque rupture or erosion.^47^ This leads to progressive myocardial ischaemia which, if sustained, leads to infarction via three possible mechanisms: (i) intraluminal platelet aggregation resulting in partial or complete vascular occlusion; (ii) release of platelet microaggregates which results in the microembolization^48^ of small vessels to cause localized ischaemia and infarction; (iii) progression of white thrombus formation to clotting cascade activation which results in partial- or total-occlusion of the epicardial artery.^47^ The rise and/or fall in cTnI/T is used to distinguish an AMI from unstable angina, whilst the ST-segment of the ECG is used to distinguish between STEMIs and NSTEMIs.^3^

### 5.2 The 99th percentile

**Figure 3:**
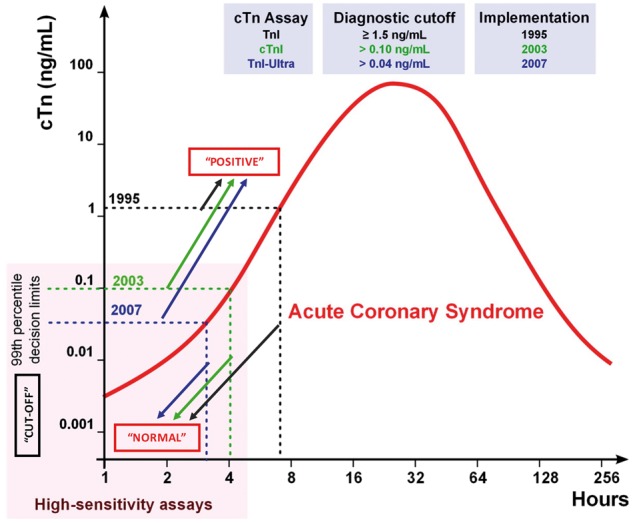
*** ***The 99th percentile diagnostic cut-off for cardiac troponin (cTn) assays. A hypothetical case of an acute coronary syndrome is shown to illustrate the evolution of cTn assay precision and sensitivity. The diagnostic cut-off for cTnI assays in 1995 was* *≥ 1.5 ng/mL, in 2003 > 0.10 ng/mL and in 2007 > 0.04 ng/mL. The increasing sensitivity of the assays meant that very small concentrations of cTn could be detected, thus the 99th percentile decision limit had to be lowered. The 99th percentile quantitatively represents a value at which 1 person in 100 will have a false positive result.^6^ Any concentration of cTn detected within the 99th percentile decision limit suggests a ‘normal’ result. Any concentration value which falls outside this decision limit indicates a ‘positive’ cTn and substantiates a possible AMI. Edited from Mahajan and Jarolim^87^ with permission.

Early generations of the cTn assay were relatively insensitive. Troponin detected by the early-generation assays were indicative of ‘substantial’ irreversible myocardial injury and carried diagnostic value.^49^ During the past two decades however, assay sensitivity has improved to such an extent, that even biological variation of cTn in the femtomolar range in a healthy individual during a 4-h period can be detected.^50^ As cTn can be detected in the normal population, the questions arises as to what concentration of cTn would have to be exceeded to constitute a ‘positive’ troponin. This dilemma is addressed by the 99th percentile, the population based reference value determined from the normal population, established to serve as the decision value by which an AMI can be confirmed or excluded (‘clinical decision value’; CDV) (*Figure *3).^51^ It should be noted however that the 99th percentile is not a panacea. It has been challenging to determine a standardized cut-off, particularly for cTnI, owing to the number of commercial assays that are available with different characteristics and 99th percentiles. This is emphasized by a recent prospective multi-centre study, whereby the approved CDVs for two clinically available high-sensitivity (hs) assays (hs-cTnI and hs-cTnT), were shown to be significantly inconsistent; one in five patients diagnosed as having an AMI using hs-cTnT will receive a different diagnosis when hs-cTnI is used.^52^ Further, the 99th percentile concentration is profoundly influenced by the population demographics such as age and gender; currently, these factors have not been standardized.^6^ A report from 2012 highlights the magnitude of the effect that underlying demographics has on the 99th percentile.^51^

For the reasons outlined above, patients presenting with chest pain and a suspected ACS may have a ‘natural’ cTn concentration above the 99th percentile, which diminishes specificity. Similarly, because of the relatively slow increase in serum cTn after myocardial injury, many patients with an ultimate diagnosis of AMI may have a cTn concentration below the 99th percentile at presentation, limiting sensitivity. To overcome these limitations, the latest guidelines for rapid rule-out/rule-in advocate decision cut-point concentrations well below and above the 99th percentile, thereby enhancing sensitivity and specificity. The drawback is that the majority of patients lie in an intermediate ‘grey-zone’ between these decision cut-points and need further investigation.^3^ Specificity is further improved by a rise and/or fall in cTn (release kinetics) being a major criterion for the diagnosis of AMI, as recommended by current guidelines (Section 3 and 4.3).^2^

### 5.3 cTn is organ-specific, not disease-specific

The high myocardial specificity and clinical sensitivity of cTnI/T for myocardial injury is well accepted.^2^ However, claiming specificity for any particular disease is untenable. It is important to acknowledge that neither cTnI nor cTnT are exclusively released due to MI, and that they can be released both as a result of ischaemic, non-ischaemic and extra-cardiac conditions.^42^ This notion is of particular relevance to emergency department (ED) clinicians where ∼20% of patients attending the ED have elevated cTn, although the majority of these patients do not have ACS.^4^ There are a large (and ever increasing) number of acute and chronic conditions where elevated cTn can be detected in the absence of ACS or other obvious causes of significant ischaemic myocardial necrosis.^4^^,^^53^^,^^54^ The elevation of cTn in the absence of ACS is likely to be multifactorial, including myocardial ischaemia, increased wall tension and ventricular strain, direct myocyte trauma, excess catecholamines, and possibly impaired renal clearance.^4^

## 6. cTn as biomarkers of chronic disease

There have been no prospective studies to date altering patient treatment to determine whether it has an effect on troponin release. As such, controversy remains as to the precise mechanisms by which cTn can be elevated in chronic disease states including chronic heart failure (CHF), diabetes, pulmonary arterial hypertension, stable coronary artery disease (CAD), and chronic kidney disease (CKD).^4^ Our current understanding on how cTn is elevated in chronic conditions is based on clinical trials and animal models.

Indeed, cTn measured using both the conventional^55^ and hs-cTn^4^ assays have been shown to have prognostic value, suggesting cTn is a good ‘barometer of risk’ in chronically unwell patients. Notably, several studies have demonstrated that cTn increases, even within the reference range of healthy individuals, is predictive of risk.^56^ Elevated baseline hs-cTnT in low-risk, normotensive, ambulatory individuals has been shown to be predictive of developing clinical hypertension.^57^ Furthermore, temporal increases in hs-cTnT over a 6-year period have been associated with subsequent CAD, HF (both reduced and preserved ejection fraction subtypes), and death.^58^

There is an extensive literature on cTn in the context of CHF, where troponin values above the 99th percentile are associated with a worse prognosis.^59^^,^^60^ Hs-cTnT levels are higher in patients with higher NYHA classes, and strongly associated with clinical outcome and all-cause mortality.^61^ Comparable conclusions were made on hs-cTnT levels and outcome in CHF by meta-analysis.^62^ An interesting observation by Masson *et al*.^62^ was that outcome tended to improve in CHF patients with decreasing hs-cTnT levels over time.

Additionally, a recent clinical study has made some important observations. 3318 male participants with moderate hypercholesterolaemia were randomized to receive placebo or pravastatin with hs-cTnI measured at baseline and at 1 year. cTnI concentration was reduced by 13% with statin therapy, and reductions in troponin concentrations were associated with better outcome (independent of low-density lipoprotein cholesterol lowering).^63^

Importantly, troponin elevations are not only relevant in individuals with cardiovascular diseases, but are important in those with extra-cardiac diseases. Sandoval *et al.*^64^ reported that serial increases in hs-cTn levels over a 3-month period in CKD patients undergoing haemodialysis was related to a greater risk of all-cause mortality.^64^ The conclusions made by Sandoval *et al.*^64^ were based on relative change values as opposed to absolute change values, which accounts for the biomarker’s biological variability.^65^ Currently, it is unclear whether absolute, or relative changes in troponin values are better for clinical practice.^65^

**Figure 4:**
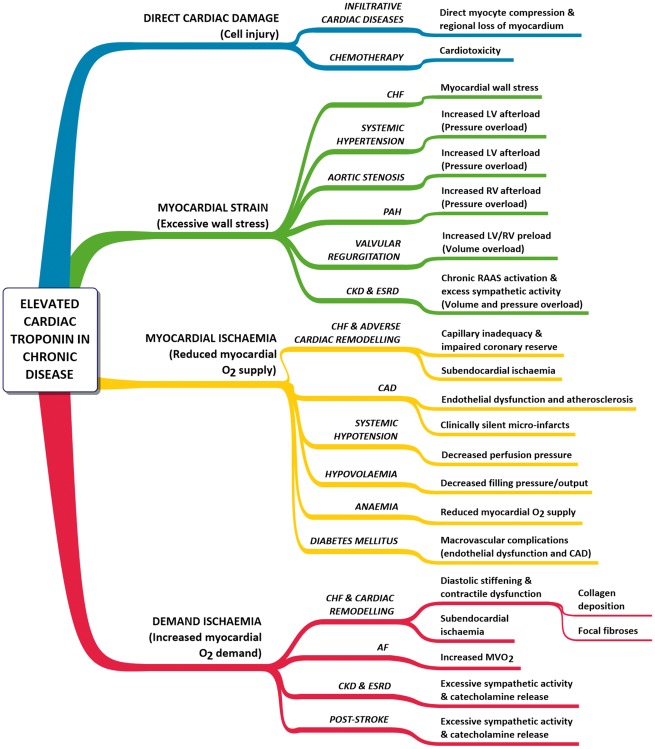
*** ***Presumed mechanisms for elevated cardiac troponin (cTn) in chronic diseases. Elevated cTn has been documented in the conditions listed. AF, atrial fibrillation; CAD, coronary artery disease; CHF, chronic heart failure; CKD, chronic kidney disease; ESRD, end-stage renal disease; LV, left ventricle; MVO_2_, myocardial oxygen consumption; PAH, pulmonary arterial hypertension; RAAS, renin-angiotensin-aldosterone system; RV, right ventricle. Adapted from Jeremias and Gibson.^53^

There are a number of chronic diseases in which elevated troponin has been documented.^53^^**,**^^54^^**,**^^59^ The possible mechanisms by which cTn can be released are illustrated in *Figure *4. Examples include diabetes mellitus, hypotension and stroke.^42^ In the case of stroke, although cTn elevations have only been reported under ‘acute’ situations (e.g. immediately following subarachnoid haemorrhage; SAH), the main risk factor for mortality long-term in stroke survivors are manifestations of cardiovascular disease (e.g. CAD).^66^ Excessive catecholamine release following SAH (demonstrated in both humans^67^ and the canine^68^) may result from cardiomyocyte death through increased myocardial demand.

Understanding these mechanisms remains a key issue and is highlighted by a large retrospective study. In a study of all patients admitted to US Veterans Affairs hospitals during 2006, only 42.8% of the patients who tested positive for cTn had a primary diagnosis of ACS.^69^ CHF and CAD were the most common diagnoses amongst these patients, but renal conditions were also a frequent diagnosis amid a range of other primary diagnoses of extra-cardiac origin. Indeed, the idea that troponin can be detected under clinical circumstances, in which there is no apparent myocardial ischaemia, is not new.^55^ What is not highlighted in *Figure *4 are the pathobiological mechanisms by which these cTn elevations can potentially occur. Broadly, there are six major potential mechanisms: myocyte necrosis, apoptosis, normal myocyte turnover, cellular release of proteolytic degradation products, increased cell wall permeability, and the formation and release of membranous blebs.^70^ Some of these mechanisms are discussed in the following sections.

To detect any biomarker, there must be either increased release of that marker, or reduced clearance from the circulation. It has been proposed that cTn detected in patients with chronic renal dysfunction is a result of reduced renal clearance. However, the evidence for this is debated and the notion of reduced clearance remains controversial (Section 6.3). The lines of evidence to date strongly support the case that we are detecting troponin that is released as opposed to that not being cleared. We will discuss key evidence and possible mechanisms by which cTn had been reported to be elevated in a subset of chronic conditions.

### 6.1 Myocardial ischaemia

During initial assay development, it was established that troponin is released exclusively upon cardiac myocyte necrosis with membrane disruption, due to irreversible cell damage.^27^^,^^28^ This was validated by an experimental study by Fishbein *et al.*^71^ The authors performed immunohistochemistry with antibodies against cTnI and cTnT, on archival, formalin-fixed, paraffin-embedded myocardium from a large number of experimental animals that had undergone different durations of coronary occlusion with/without reperfusion. Their results showed that cTnI/T can be released as early as 30 min of coronary occlusion, preceding histologic evidence of necrosis. The conclusion was that all loss of cTnI/T from myocardium was necrotic.^72^

However, it was postulated that myocardial ischaemia, without necrosis, can result in the release of troponin.^73^ Clinical studies have reported increased cTn without any obvious ischaemic heart disease,^74^ and also, after extreme exercise^75^ (although the intensity and modality of exercise undertaken may also be a determinant in cTn release).^76^^,^^77^ As reported by several authors,^4^^,^^70^^,^^75^ the suggestion has been made that troponin can be released ‘without irreversible necrosis’ or with ‘reversible cell damage’, leading to debate as to whether myocyte necrosis (or even myocyte death) is necessary for troponin release.^75^ Despite a number of interesting postulates, currently accepted experimental evidence supports that the release, and detection of cTn, is due to irreversible cell death.^33^ Some of the experimental evidence which has led to the possible view that cTn may be detected with chronic ischaemia alone, without necrosis, is discussed below.

Stable CAD has been implicated as a condition associated with elevated cTn and it is thought that repeated, short-lived episodes of ischaemia over a ‘chronic’ time-course, could be part of its aetiology.^4^ However, there are no experimental studies that have aimed to directly address this. Therefore, to establish how CAD may result in elevated cTn, we are restricted to causal relationships established from clinical studies and to inferring mechanisms from experimental studies using myocardial ischaemia. Feng *et al.*^78^ showed that reduced flow in the left anterior descending artery (LAD; as a mimic of severe coronary artery stenosis; ∼36% reduction in flow) in an *in vivo* swine model resulted in elevation of cTnI, CK-MB and myoglobin. Hearts were subsequently excised and histologically examined (triphenyltetrazolium chloride staining) to assess whether infarction was evident. Pigs that did not develop necrosis (assessed by histology) still released cTnI. The authors concluded that increased levels of cTnI can be detected after reversible and irreversible myocardial ischaemic injury.

Elevated cTn in stable CAD may be attributed to enhanced proteolytic degradation of myofibrillar troponin. Reduced Ca^2+^ extrusion by the Na/Ca exchanger, with concomitant reduced Ca^2+^ uptake by the sarcoplasmic reticulum, may produce local elevations of the concentration of intracellular Ca^2+^ during ischaemia.^79^ With repeated episodes of short-lived ischaemia (i.e. CAD), the activity of the Ca^2+^-dependent protease calpain could be increased, leading to chronic proteolytic degradation of myofibrillar cTn. However, one of the determinants which would confirm this mechanism would be whether there is increased intracellular Ca^2+^ during episodic bouts of ischaemia over chronic periods. This has not yet been studied. Indeed, in agreement with this hypothesis, it may be possible that proteolysis creates small fragments of troponin that can pass through the cellular membrane with normal membrane integrity (i.e. cTn release without cell death).^70^

Another pathobiological mechanism by which intact troponin may be released during ischaemia without necrosis is the formation and release of membranous blebs.^70^ Blebs are spherical protrusions of the plasma membrane. Their development is driven by increases in cytoplasmic (intracellular) hydrostatic pressure.^80^ Blebbing is considered to be a key characteristic of the execution phase of apoptosis, but strikingly it is also well known that blebbing is involved in key physiological processes of healthy cells such as during cytokinesis.^80^^,^^81^ It has been proposed by Hickman *et al.*^82^ that ischaemic cardiomyocytes form and release blebs containing the unbound, cytosolic faction, or the loosely-bound myofibrillar faction, of cTn. It may be entirely possible that cardiomyocytes form and release blebs. However, although well established in hepatocytes, the evidence for blebbing in cardiomyocytes has not been substantiated.

A recent experimental study using an *in vivo* swine model demonstrated cTnI release following 10 min of coronary occlusion.^83^ Although histological evidence of necrosis was absent after the brief period of ischaemia, positive caspase staining (Section 4.4) and terminal deoxynucleotidyl transferase-mediated dUTP nick-end labelling (TUNEL) suggested myocytes underwent irreversible injury from apoptosis.^83^ The precise mechanism of cell death is debateable, since apoptotic bodies, in principle, retain membrane integrity. Although a possibility may be that cardiomyocyte apoptosis transitioned to secondary necrosis,^84^ alternative mechanisms could exist.^85^

Collectively, it is important these concepts be interpreted carefully. Indeed, there is a spectrum of injury with myocardial ischaemia/reperfusion, ranging from reversible damage with mild ischaemia (where there is functional recovery), to irreversible injury with severe ischaemia.^86^ However, although there may be no overt evidence of cell death at the organ level, it is highly unlikely even a small population of myocytes have not died.^70^ Considering that the analytical sensitivity of conventional cTn assays are in the picomolar range, whilst hs-cTn assays can detect cTn in the femtomolar range,^87^ what is more likely the case is that there is death of cardiomyocytes, but at a level which cannot be detected by any alternative analytical method (e.g. triphenyltetrazolium chloride, lactate elution). A new experimental study substantiates this idea, whereby hs-cTn assays were able to detect serum elevations of troponin from necrosis of a few milligrams of myocardium: an amount of irreversible injury beyond the resolution of any imaging technique.^88^

### 6.2 Myocardial strain

The idea of ‘myocardial strain’ describes the release of cTn due to cardiomyocyte injury resulting from mechanical deformation or physiological stress.^53^ During CHF, there is ventricular volume and pressure overload. This was hypothesized by Jeremias and Gibson^53^ to cause excessive wall tension and direct myofibrillar damage, resulting in cardiomyocyte death, and thus troponin release in the absence of ischaemia (myocardial strain theory). This is supported by both experimental studies and clinical observations.

Cheng *et al.*^89^ demonstrated in an *in vitro* study, stretch-mediated apoptosis of papillary muscle myocytes. Their data showed excessive stretch can result in apoptosis, which in the context of our discussion, may result in cTn release. Indeed, detection of cTn would be dependent on whether apoptotic cells lose membrane integrity.^84^

In a clinical study by Logeart *et al.*,^90^ left-ventricular (LV) assessment was performed in conjunction with measuring cTnI and B-type natriuretic peptide (BNP) concentrations (a marker of ventricular wall strain) in 71 CHF patients. Patients with CAD and atherosclerotic abnormalities were excluded. They made the following observations: (i) there is a positive correlation between cTnI concentration and both LV wall thickness and BNP levels; (ii) cTnI release occurs in patients without ischaemic disease. Combining these findings and those of previous studies where BNP levels and LV filling pressure were positively correlated, the authors postulated that the release of cTnI could be due to significantly high LV filling pressures, causing stretch-mediated cardiomyocyte death. The authors also confirmed by imaging that these patients had increased wall thickness, which may have resulted in endocardial ischaemia and cardiomyocyte death.

Increased preload (diastolic wall stress) is a key feature of the failing heart. Both clinical and experimental studies suggest it may initiate troponin release. In an elegant clinical study, Takashio *et al.*^91^ measured the Δhs-cTnT level between the aortic root and the coronary sinus, in 76 CHF patients undergoing cardiac catheterization. This approach was taken to exclude alternative clinical causes of troponin release (e.g. CAD). The aetiology of HF in these patients was non-ischaemic (e.g. cardiomyopathy). The authors found that Δhs-cTnT was higher in HF patients vs. non-HF patients (*n* = 28), and that Δhs-cTnT was significantly correlated to LV end-diastolic pressure (a primary determinant of preload).

In Langendorff-perfused rat hearts, high preload resulted in greater degradation of cTnI: an effect attenuated by adding calpeptin (a calpain inhibitor). As a result, Feng *et al.*^92^ demonstrated that increased preload may result in increased myocyte Ca^2+^-entry secondary to mechanical strain independent of ischaemia, thus leading to activation of μ-calpains and calpain-mediated cTnI proteolysis. As an aside, results from this study have been used to theorize the release of cTn during acute decompensated HF.^4^ It should be noted however, results from Feng *et al*.^92^ have been interpreted by some investigators as the release of troponin by live, viable cardiomyocytes which exhibit increased sarcolemmal permeability, thus enabling troponin ‘leakage’.^70^ In other words, no cell death. Furthermore, it has been suggested that intact cTn may be released by live, viable myocytes, via stretch-mediated integrin stimulation. This has been reported in cultured neonatal rat cardiomyocytes^93^ where peptide-mediated integrin agonism was shown to result in increased cTnI release in the absence of necrosis [assessed by LDH assays and nuclear propidium iodide staining].

Jaffe and Wu^75^ identify experimental issues with interpreting the findings from Hessel *et al*.^93^ (e.g. the use of LDH as a biomarker of cell death). However, these issues aside, there is some simple physiology to consider when thinking about the idea of increased membrane permeability. Troponins are large molecular weight proteins (cTnI ∼24 kDa; cTnT ∼37 kDa), and there is currently, no specialized biological process (e.g. exocytosis) that has been validated for the physiological, or pathological release of these large proteins. Moreover, should there be an increase in cardiomyocyte membrane permeability, to the extent at which free troponin from the cytosol or loosely-bound myofibrillar troponin can freely leave the myocyte, the possibility cannot be ignored that Ca^2+^ will leak into the myocyte following a transmembrane concentration gradient (causing hypercontracture and Ca^2+^-mediated cell death).

It is clear that at present, as highlighted by Jaffe and Wu,^75^ although necrosis is not a requisite for cTn release, cell death in any shape or form can result in its release (e.g. autophagy, apoptosis, necroptosis).^94^ Altogether, the mechanistic and clinical studies discussed provide some compelling data indicating that cTn can be released due to cell death caused by ‘strain’–most likely via proteolytic and apoptotic mechanisms.

### 6.3 Chronic kidney disease

Elevated cTnI and cTnT are frequently observed in patients with CKD.^95^ It has been shown in a meta-analysis that cTnT in end-stage renal disease (ESRD) carries prognostic significance, with elevated cTnT being strongly associated with mortality.^96^ Increases in hs-cTnI levels have been demonstrated to be predictive for sudden cardiac death: a frequent and major cause of mortality in CKD/ESRD patients.^64^ However, with limited and often contradictory experimental data available, the mechanism(s) by which cTn is elevated in CKD is poorly understood.^97^ Data from clinical studies strongly suggest direct cardiac damage in the absence of acute myocardial ischaemia to be the mechanism of increased cTn release.^95^ With a high incidence of CAD in CKD patients^98^ such damage may be attributable to clinically-silent micro-infarcts (i.e. subclinical cardiac damage).^99^ LV hypertrophy and raised LV preload are common in patients with ESRD, which could lead to increased cTn release as a result of myocardial strain and myocardial O_2_ supply-demand mismatch.^100^ CKD patients are also typically hypertensive.^98^ Thus myocardial strain consequent to increased LV afterload could present another mechanism by which cTn is elevated.

Reaching a consensus on the mechanisms by which cTn is elevated in CKD and ESRD has been complicated by the multiple assay platforms available, but also by the fact that the stage of renal disease is not standardized across studies. Most of the evidence available has been on ESRD patients undergoing regular haemodialysis. In such patients, cTnT is more frequently elevated than cTnI.^101^ An explanation for this observation may be that cTnI, but not cTnT, adheres to polysulphone dialyser membranes during haemodialysis.^102^ Indeed, polysulphone membranes are not the only types in current clinical use, but it does raise the interesting question that cTnI may be adhering to other types of synthetic dialysis membrane. The mechanisms by which cTn is detected in CKD and ESRD patients remains highly controversial.

## 7. Conclusions

Originally the rationale behind the cTn assay was relatively simple: myocardial necrosis leads to membrane disruption causing troponin release which is detected in serum. The troponins have been used to diagnose acute myocardial injury and such use has become engrained in the Universal Definition of Acute Myocardial Infarction. Today however, with the evolving sensitivity of cTn assays, it is clear cTn is detectable in everyone and becomes elevated above the 99th percentile in stable chronic conditions. These features of the high-sensitivity assays have made the interpretation of cTn results more complex.

In recent years, the concept that troponin can be released with reversible cell injury, without necrosis, or even cell death, has been repeatedly suggested. In part, this is due to increased cTn being observed in several clinical situations whereby there are no obvious signs of overt cardiac disease, and in particular with the consistent finding of increased hs-cTn following extreme exercise. However, it is emphasized that current evidence reinforces the view that cTn is only released from cardiomyocytes upon irreversible cell death (whether it be by necrosis or apoptosis etc.).

Future research needs to embrace the high-sensitivity of the latest assays to expand their use in personalizing medical therapy. In particular, we believe that concentrations below and around the 99th percentile could be used to select higher risk patients for future randomized trials in HF and prevention of vascular events. Another under explored area is understanding if additional information, over and above concentration, is gained by measurement of post-translational modifications in circulating cTnI and cTnT. Since varied forms of cTn can be detected in serum following AMI (e.g. following proteolytic cleavage, post-translational modifications etc.), the future assays may not just feature enhanced analytic sensitivity, but also the ability to detect different forms of cTn released during different ‘stages‘of ischaemia–as identified by Wu *et al.*^35^ and proposed by McDonough and Van Eyk.^45^ Whatever the direction of evolution, one thing is certain: biomarkers of myocardial injury are here to stay.

## References

[1] Greaser ML, Gergely J. Purification and properties of the components from troponin. J Biol Chem 1973;248:2125–2133. 4266138

[2] Thygesen K, Alpert JS, Jaffe AS, Simoons ML, Chaitman BR, White HD, Thygesen K, Alpert JS, White HD, Jaffe AS, Katus HA, Apple FS, Lindahl B, Morrow DA, Chaitman BA, Clemmensen PM, Johanson P, Hod H, Underwood R, Bax JJ, Bonow RO, Pinto F, Gibbons RJ, Fox KA, Atar D, Newby LK, Galvani M, Hamm CW, Uretsky BF, Steg PG, Wijns W, Bassand JP, Menasche P, Ravkilde J, Ohman EM, Antman EM, Wallentin LC, Armstrong PW, Simoons ML, Januzzi JL, Nieminen MS, Gheorghiade M, Filippatos G, Luepker RV, Fortmann SP, Rosamond WD, Levy D, Wood D, Smith SC, Hu D, Lopez-Sendon JL, Robertson RM, Weaver D, Tendera M, Bove AA, Parkhomenko AN, Vasilieva EJ, Mendis S. Joint ESC/ACCF/AHA/WHF Task Force for the Universal Definition of Myocardial Infarction. Third universal definition of myocardial infarction. Eur Heart J 2012;33:2551–2567.22922414

[3] Roffi M, Patrono C, Collet J-P, Mueller C, Valgimigli M, Andreotti F, Bax JJ, Borger MA, Brotons C, Chew DP, Gencer B, Hasenfuss G, Kjeldsen K, Lancellotti P, Landmesser U, Mehilli J, Mukherjee D, Storey RF, Windecker S, Baumgartner H, Gaemperli O, Achenbach S, Agewall S, Badimon L, Baigent C, Bueno H, Bugiardini R, Carerj S, Casselman F, Cuisset T, Erol Ç, Fitzsimons D, Halle M, Hamm C, Hildick-Smith D, Huber K, Iliodromitis E, James S, Lewis BS, Lip GYH, Piepoli MF, Richter D, Rosemann T, Sechtem U, Steg PG, Vrints C, Luis Zamorano J. 2015 ESC Guidelines for the management of acute coronary syndromes in patients presenting without persistent ST-segment elevation: Task Force for the Management of Acute Coronary Syndromes in Patients Presenting without Persistent ST-Segment Elevation of the European Society of Cardiology (ESC). Eur Heart J 2016;37:267–315. 26320110 10.1093/eurheartj/ehv320

[4] Giannitsis E, Katus HA. Cardiac troponin level elevations not related to acute coronary syndromes. Nat Rev Cardiol 2013;10:623–634. 23979214 10.1038/nrcardio.2013.129

[5] Tobacman LS. Thin filament-mediated regulation of cardiac contraction. Annu Rev Physiol 1996;58:447–481. 8815803 10.1146/annurev.ph.58.030196.002311

[6] Baker JO, Reinhold J, Redwood S, Marber MS. Troponins: redefining their limits. Heart 2011;97:447–452. 21193685 10.1136/hrt.2010.205617

[7] Hartshorne DJ, Mueller H. Fractionation of troponin into two distinct proteins. Biochem Biophys Res Commun 1968;31:647–653. 4233121 10.1016/0006-291x(68)90610-4

[8] Greaser ML, Gergely J. Reconstitution of troponin activity from three protein components. J Biol Chem 1971;246:4226–4233. 4253596

[9] Collinson PO, Boa FG, Gaze DC. Measurement of cardiac troponins. Ann Clin Biochem 2001;38:423–449. 11587122 10.1177/000456320103800501

[10] Farah CS, Reinach FC. The troponin complex and regulation of muscle contraction. Faseb J 1995;9:755–767. 7601340 10.1096/fasebj.9.9.7601340

[11] Parmacek MS, Solaro RJ. Biology of the troponin complex in cardiac myocytes. Prog Cardiovasc Dis 2004;47:159–176. 15736582 10.1016/j.pcad.2004.07.003

[12] Hunkeler NM, Kullman J, Murphy AM. Troponin I isoform expression in human heart. Circ Res 1991;69:1409–1414. 1934363 10.1161/01.res.69.5.1409

[13] Sasse S, Brand NJ, Kyprianou P, Dhoot GK, Wade R, Arai M, Periasamy M, Yacoub MH, Barton PJ. Troponin I gene expression during human cardiac development and in end-stage heart failure. Circ Res 1993;72:932–938. 8477526 10.1161/01.res.72.5.932

[14] Anderson PA, Malouf NN, Oakeley AE, Pagani ED, Allen PD. Troponin T isoform expression in humans. A comparison among normal and failing adult heart, fetal heart, and adult and fetal skeletal muscle. Circ Res 1991;69:1226–1233. 1934353 10.1161/01.res.69.5.1226

[15] Nadal-Ginard B, Mahdavi V. Molecular basis of cardiac performance. Plasticity of the myocardium generated through protein isoform switches. J Clin Invest 1989;84:1693–1700. 2687327 10.1172/JCI114351 PMC304044

[16] Thygesen K, Mair J, Katus H, Plebani M, Venge P, Collinson P, Lindahl B, Giannitsis E, Hasin Y, Galvani M, Tubaro M, Alpert JS, Biasucci LM, Koenig W, Mueller C, Huber K, Hamm C, Jaffe AS. Recommendations for the use of cardiac troponin measurement in acute cardiac care. Eur Heart J 2010;31:2197–2204. 20685679 10.1093/eurheartj/ehq251

[17] Jaffe AS, Vasile VC, Milone M, Saenger AK, Olson KN, Apple FS. Diseased skeletal muscle: a noncardiac source of increased circulating concentrations of cardiac troponin T. J Am Coll Cardiol 2011;58:1819–1824. 21962825 10.1016/j.jacc.2011.08.026

[18] Nishii K, Morimoto S, Minakami R, Miyano Y, Hashizume K, Ohta M, Zhan DY, Lu QW, Shibata Y. Targeted disruption of the cardiac troponin T gene causes sarcomere disassembly and defects in heartbeat within the early mouse embryo. Dev Biol 2008;322:65–73. 18671960 10.1016/j.ydbio.2008.07.007

[19] Huang X, Pi Y, Lee KJ, Henkel AS, Gregg RG, Powers PA, Walker JW. Cardiac troponin I gene knockout: a mouse model of myocardial troponin I deficiency. Circ Res 1999;84:1–8. 9915769 10.1161/01.res.84.1.1

[20] Grand RJA, Wilkinson JM, Mole LE. The amino acid sequence of rabbit cardiac troponin I. Biochem J 1976;159:633–641. 1008822 10.1042/bj1590633 PMC1164163

[21] Pearlstone JR, Carpenter MR, Smillie LB. Amino acid sequence of rabbit cardiac troponin T. J Biol Chem 1986;261:16795–16810. 3782144

[22] Vallins WJ, Brand NJ, Dabhade N, Butler-Browne G, Yacoub MH, Barton PJR. Molecular cloning of human cardiac troponin I using polymerase chain reaction. FEBS Lett 1990;270:57–61. 2226790 10.1016/0014-5793(90)81234-f

[23] Townsend PJ, Farza H, MacGeoch C, Spurr NK, Wade R, Gahlmann R, Yacoub MH, Barton PJ. Human cardiac troponin T: identification of fetal isoforms and assignment of the TNNT2 locus to chromosome 1q. Genomics 1994;21:311–316. 8088824 10.1006/geno.1994.1271

[24] Alpert JS, Thygesen K, Antman EM, Bassand JP. Myocardial infarction redefined - A consensus document of The Joint ESC/ACC Committee for the redefinition of myocardial infarction. Eur Heart J 2000;21:1502–1513. 10973764 10.1053/euhj.2000.2305

[25] Collinson PO, Garrison L, Christenson RH. Cardiac biomarkers - A short biography. Clin Biochem 2015;48:197–200. 25464015 10.1016/j.clinbiochem.2014.11.014

[26] Bernard R, Corday E, Eliasch H, Gonin A. Nomenclature and criteria for diagnosis of ischaemic heart disease. Circulation 1979;59:607–609. 761341 10.1161/01.cir.59.3.607

[27] Cummins B, Auckland ML, Cummins P. Cardiac-specific troponin-I radioimmunoassay in the diagnosis of acute myocardial infarction. Am Heart J 1987;113:1333–1344. 3591601 10.1016/0002-8703(87)90645-4

[28] Katus HA, Remppis A, Looser S, Hallermeier K, Scheffold T, Kubler W. Enzyme linked immuno assay of cardiac troponin T for the detection of acute myocardial infarction in patients. J Mol Cell Cardiol 1989;21:1349–1353. 2632816 10.1016/0022-2828(89)90680-9

[29] Wu AH, Apple FS, Gibler WB, Jesse RL, Warshaw MM, Valdes R Jr, National Academy of Clinical Biochemistry Standards of Laboratory Practice: recommendations for the use of cardiac markers in coronary artery diseases. Clin Chem 1999;45:1104–1121. 10388496

[30] Thygesen K, Alpert JS, White HD, Jaffe AS, Apple FS, Galvani M, Katus HA, Newby LK, Ravkilde J, Chaitman B, Clemmensen PM, Dellborg M, Hod H, Porela P, Underwood R, Bax JJ, Beller GA, Bonow R, Van Der Wall EE, Bassand J-P, Wijns W, Ferguson TB, Steg PG, Uretsky BF, Williams DO, Armstrong PW, Antman EM, Fox KA, Hamm CW, Ohman EM, Simoons ML, Poole-Wilson PA, Gurfinkel EP, Lopez-Sendon J-L, Pais P, Mendis S, Zhu J-R, Wallentin LC, Fernandez-Aviles F, Fox KM, Parkhomenko AN, Priori SG, Tendera M, Voipio-Pulkki L-M, Vahanian A, Camm AJ, De Caterina R, Dean V, Dickstein K, Filippatos G, Funck-Brentano C, Hellemans I, Kristensen SD, McGregor K, Sechtem U, Silber S, Tendera M, Widimsky P, Zamorano JL, Morais J, Brener S, Harrington R, Morrow D, Sechtem U, Lim M, Martinez-Rios MA, Steinhubl S, Levine GN, Gibler WB, Goff D, Tubaro M, Dudek D, Al-Attar N. Joint ESC/ACCF/AHA/WHF Task Force for the Redefinition of Myocardial Infarction. Universal definition of myocardial infarction. Eur Heart J 2007;28:2525–2538. 17951287 10.1093/eurheartj/ehm355

[31] Melanson SE, Tanasijevic MJ, Jarolim P. Cardiac troponin assays: a view from the clinical chemistry laboratory. Circulation 2007;116:e501–e504. 17967982 10.1161/CIRCULATIONAHA.107.722975

[32] Amundson BE, Apple FS. Cardiac troponin assays: a review of quantitative point-of-care devices and their efficacy in the diagnosis of myocardial infarction. Clin Chem Lab Med 2015;53:665–676. 25324453 10.1515/cclm-2014-0837

[33] Apple FS, Collinson PO. Analytical characteristics of high-sensitivity cardiac troponin assays. Clin Chem 2012;58:54–61. 21965555 10.1373/clinchem.2011.165795

[34] Gaze DC, Collinson PO. Multiple molecular forms of circulating cardiac troponin: analytical and clinical significance. Ann Clin Biochem 2008;45:349–355. 18583618 10.1258/acb.2007.007229

[35] Wu AH, Feng YJ, Moore R, Apple FS, McPherson PH, Buechler KF, Bodor G. Characterization of cardiac troponin subunit release into serum after acute myocardial infarction and comparison of assays for troponin T and I. Clin Chem 1998;44:1198–1208. 9625043

[36] Jaffe AS, Babuin L, Apple FS. Biomarkers in acute cardiac disease: the present and the future. J Am Coll Cardiol 2006;48:1–11. 16814641 10.1016/j.jacc.2006.02.056

[37] Yellon DM, Hausenloy DJ. Myocardial reperfusion injury. N Engl J Med 2007;357:1121–1135. 17855673 10.1056/NEJMra071667

[38] Heusch G, Gersh BJ. The pathophysiology of acute myocardial infarction and strategies of protection beyond reperfusion: a continual challenge. Eur Heart J 2017;38:774–784. 27354052 10.1093/eurheartj/ehw224

[39] Katus HA, Remppis A, Scheffold T, Diederich KW, Kuebler W. Intracellular compartmentation of cardiac troponin T and its release kinetics in patients with reperfused and nonreperfused myocardial infarction. Am J Cardiol 1991;67:1360–1367. 1904190 10.1016/0002-9149(91)90466-x

[40] Bleier J, Vorderwinkler KP, Falkensammer J, Mair P, Dapunt O, Puschendorf B, Mair J. Different intracellular compartmentations of cardiac troponins and myosin heavy chains: a causal connection to their different early release after myocardial damage. Clin Chem 1998;44:1912–1918. 9732976

[41] Remppis A, Scheffold T, Greten J, Haass M, Greten T, Kubler W, Katus HA. Intracellular compartmentation of troponin T: release kinetics after global ischemia and calcium paradox in the isolated perfused rat heart. J Mol Cell Cardiol 1995;27:793–803. 7776386 10.1016/0022-2828(95)90086-1

[42] Agewall S, Giannitsis E, Jernberg T, Katus H. Troponin elevation in coronary vs. non-coronary disease. Eur Heart J 2011;32:404–411b. 21169615 10.1093/eurheartj/ehq456

[43] Vasile VC, Jaffe AS. The biological basis of troponin in heart disease: possible uses for troponin fragmentology. Heart Metab 2009;43:5–8.

[44] Nishida K, Yamaguchi O, Otsu K. Degradation systems in heart failure. J Mol Cell Cardiol 2015;84:212–222. 25981331 10.1016/j.yjmcc.2015.05.004

[45] McDonough JL, Van Eyk JE. Developing the next generation of cardiac markers: disease-induced modifications of troponin I. Prog Cardiovasc Dis 2004;47:207–216. 15736586 10.1016/j.pcad.2004.07.001

[46] Heusch G. Myocardial ischemia: lack of coronary blood flow or myocardial oxygen supply/demand imbalance? Circ Res 2016;119:194–196. 27390331 10.1161/CIRCRESAHA.116.308925

[47] Collinson PO, Gaze DC. Biomarkers of cardiovascular damage. Med Princ Pract 2007;16:247–261. 17541289 10.1159/000102146

[48] Heusch G, Kleinbongard P, Bose D, Levkau B, Haude M, Schulz R, Erbel R. Coronary microembolization: from bedside to bench and back to bedside. Circulation 2009;120:1822–1836. 19884481 10.1161/CIRCULATIONAHA.109.888784

[49] Jesse RL. On the relative value of an assay versus that of a test: a history of troponin for the diagnosis of myocardial infarction. J Am Coll Cardiol 2010;55:2125–2128. 20447536 10.1016/j.jacc.2010.03.014

[50] Wu AH, Lu QA, Todd J, Moecks J, Wians F. Short- and long-term biological variation in cardiac troponin I measured with a high-sensitivity assay: implications for clinical practice. Clin Chem 2009;55:52–58. 18988755 10.1373/clinchem.2008.107391

[51] Collinson PO, Heung YM, Gaze D, Boa F, Senior R, Christenson R, Apple FS. Influence of population selection on the 99th percentile reference value for cardiac troponin assays. Clin Chem 2012;58:219–225. 22100808 10.1373/clinchem.2011.171082

[52] Wildi K, Gimenez MR, Twerenbold R, Reichlin T, Jaeger C, Heinzelmann A, Arnold C, Nelles B, Druey S, Haaf P, Hillinger P, Schaerli N, Kreutzinger P, Tanglay Y, Herrmann T, Moreno Weidmann Z, Krivoshei L, Freese M, Stelzig C, Puelacher C, Rentsch K, Osswald S, Mueller C. Misdiagnosis of myocardial infarction related to limitations of the current regulatory approach to define clinical decision values for cardiac troponin. Circulation 2015;131:2032–2040. 25948541 10.1161/CIRCULATIONAHA.114.014129 PMC4456170

[53] Jeremias A, Gibson CM. Alternative causes for elevated cardiac troponin levels when acute coronary syndromes are excluded. Ann Intern Med 2005;142:786–791. 15867411 10.7326/0003-4819-142-9-200505030-00015

[54] Newby LK, Jesse RL, Babb JD, Christenson RH, De Fer TM, Diamond GA, Fesmire FM, Geraci SA, Gersh BJ, Larsen GC, Kaul S, McKay CR, Philippides GJ, Weintraub WS, Harrington RA, Bhatt DL, Anderson JL, Bates ER, Bridges CR, Eisenberg MJ, Ferrari VA, Fisher JD, Garcia MJ, Gardner TJ, Gentile F, Gilson MF, Hernandez AF, Hlatky MA, Jacobs AK, Kaul S, Linderbaum JA, Moliterno DJ, Mukherjee D, Rosenson RS, Stein JH, Weitz HH, Wesley DJ. ACCF 2012 expert consensus document on practical clinical considerations in the interpretation of troponin elevations. J Am Coll Cardiol 2012;60:2427–2463. 23154053 10.1016/j.jacc.2012.08.969

[55] Collinson PO, Stubbs PJ. Are troponins confusing? Heart 2003;89:1285–1287. 14594878 10.1136/heart.89.11.1285 PMC1767954

[56] Collinson PO. The role of cardiovascular biomarkers in cardiovascular disease risk assessment. Curr Opin Cardiol 2014;29:366–371. 24848409 10.1097/HCO.0000000000000081

[57] McEvoy JW, Chen Y, Nambi V, Ballantyne CM, Sharrett AR, Appel LJ, Post WS, Blumenthal RS, Matsushita K, Selvin E. High-sensitivity cardiac troponin t and risk of hypertension. Circulation 2015;132:825–833. 26152706 10.1161/CIRCULATIONAHA.114.014364 PMC4558242

[58] McEvoy JW, Chen Y, Ndumele CE, Solomon SD, Nambi V, Ballantyne CM, Blumenthal RS, Coresh J, Selvin E. Six-year change in high-sensitivity cardiac troponin t and risk of subsequent coronary heart disease, heart failure, and death. JAMA Cardiol 2016;1:519–528. 27439107 10.1001/jamacardio.2016.0765 PMC5084093

[59] Januzzi JL Jr, Filippatos G, Nieminen M, Gheorghiade M. Troponin elevation in patients with heart failure: on behalf of the third Universal Definition of MI Global Task Force. Eur Heart J 2012;33:2265–2271. 22745356 10.1093/eurheartj/ehs191

[60] Liquori ME, Christenson RH, Collinson PO, Defilippi CR. Cardiac biomarkers in heart failure. Clin Biochem 2014;47:327–337. 24530339 10.1016/j.clinbiochem.2014.01.032

[61] Tentzeris I, Jarai R, Farhan S, Perkmann T, Schwarz MA, Jakl G, Wojta J, Huber K. Complementary role of copeptin and high-sensitivity troponin in predicting outcome in patients with stable chronic heart failure. Eur J Heart Fail 2011;13:726–733. 21616953 10.1093/eurjhf/hfr049

[62] Masson S, Anand I, Favero C, Barlera S, Vago T, Bertocchi F, Maggioni AP, Tavazzi L, Tognoni G, Cohn JN, Latini R. Serial measurement of cardiac troponin T using a highly sensitive assay in patients with chronic heart failure: data from 2 large randomized clinical trials. Circulation 2012;125:280–288. 22139751 10.1161/CIRCULATIONAHA.111.044149

[63] Ford I, Shah ASV, Zhang R, McAllister DA, Strachan FE, Caslake M, Newby DE, Packard CJ, Mills NL. High-sensitivity cardiac troponin, statin therapy, and risk of coronary heart disease. J Am Coll Cardiol 2016;68:2719–2728. 28007133 10.1016/j.jacc.2016.10.020 PMC5176330

[64] Sandoval Y, Herzog CA, Love SA, Cao J, Hu Y, Wu AH, Gilbertson D, Brunelli SM, Young A, Ler R, Apple FS. Prognostic value of serial changes in high-sensitivity cardiac troponin I and T over 3 months using reference change values in hemodialysis patients. Clin Chem 2016;62:631–638. 26847217 10.1373/clinchem.2015.251835

[65] Wu AH. Biological and analytical variation of clinical biomarker testing: implications for biomarker-guided therapy. Curr Heart Fail Rep 2013;10:434–440. 24037380 10.1007/s11897-013-0156-6

[66] Dixit S, Castle M, Velu RP, Swisher L, Hodge C, Jaffe AS. Cardiac involvement in patients with acute neurologic disease: confirmation with cardiac troponin I. Arch Intern Med 2000;160:3153–3158. 11074746 10.1001/archinte.160.20.3153

[67] Banki NM, Kopelnik A, Dae MW, Miss J, Tung P, Lawton MT, Drew BJ, Foster E, Smith W, Parmley WW, Zaroff JG. Acute neurocardiogenic injury after subarachnoid hemorrhage. Circulation 2005;112:3314–3319. 16286583 10.1161/CIRCULATIONAHA.105.558239

[68] Masuda T, Sato K, Yamamoto S, Matsuyama N, Shimohama T, Matsunaga A, Obuchi S, Shiba Y, Shimizu S, Izumi T. Sympathetic nervous activity and myocardial damage immediately after subarachnoid hemorrhage in a unique animal model. Stroke 2002;33:1671–1676. 12053010 10.1161/01.str.0000016327.74392.02

[69] McFalls EO, Larsen G, Johnson GR, Apple FS, Goldman S, Arai A, Nallamothu BK, Jesse R, Holmstrom ST, Sinnott PL. Outcomes of hospitalized patients with non-acute coronary syndrome and elevated cardiac troponin level. Am J Med 2011;124:630–635. 21601821 10.1016/j.amjmed.2011.02.024 PMC3771399

[70] White HD. Pathobiology of troponin elevations: do elevations occur with myocardial ischemia as well as necrosis? J Am Coll Cardiol 2011;57:2406–2408. 21658560 10.1016/j.jacc.2011.01.029

[71] Fishbein MC, Wang T, Matijasevic M, Hong L, Apple FS. Myocardial tissue troponins T and I. An immunohistochemical study in experimental models of myocardial ischemia. Cardiovasc Pathol 2003;12:65–71. 12684160 10.1016/s1054-8807(02)00188-6

[72] Hessel MH, Michielsen EC, Atsma DE, Schalij MJ, van der Valk EJ, Bax WH, Hermens WT, van Dieijen-Visser MP, van der Laarse A. Release kinetics of intact and degraded troponin I and T after irreversible cell damage. Exp Mol Pathol 2008;85:90–95. 18721805 10.1016/j.yexmp.2008.07.002

[73] Wu AH, Ford L. Release of cardiac troponin in acute coronary syndromes: ischemia or necrosis? Clin Chim Acta 1999;284:161–174. 10451243 10.1016/s0009-8981(99)00078-9

[74] Mohlenkamp S, Leineweber K, Lehmann N, Braun S, Roggenbuck U, Perrey M, Broecker-Preuss M, Budde T, Halle M, Mann K, Jockel KH, Erbel R, Heusch G. Coronary atherosclerosis burden, but not transient troponin elevation, predicts long-term outcome in recreational marathon runners. Basic Res Cardiol 2014;109:391. 24253174 10.1007/s00395-013-0391-8

[75] Jaffe AS, Wu AH. Troponin release - reversible or irreversible injury? Should we care? Clin Chem 2012;58:148–150. 22039010 10.1373/clinchem.2011.173070

[76] Gresslien T, Agewall S. Troponin and exercise. Int J Cardiol 2016;221:609–621. 27420587 10.1016/j.ijcard.2016.06.243

[77] Mazzeschi C, Piana N, Capezzali D, Mommi A, Aiello C, Gatti M, Romani G, Buratta L, Battistini D, Nasini G, Reginato E, Urbani L, Pazzagli C, Ferri C, Ambrosio G, De Feo P. The impact of strenuous group physical activity on mood states, personal views, body composition, and markers of myocardial damage in overweight/obese adults: the “Step-by-step Italy's coast to coast” trek. Biomed Res Int 2014;2014:854129. 25143947 10.1155/2014/854129 PMC4131115

[78] Feng YJ, Chen C, Fallon JT, Lai T, Chen L, Knibbs DR, Waters DD, Wu AH. Comparison of cardiac troponin I, creatine kinase-MB, and myoglobin for detection of acute ischemic myocardial injury in a swine model. Am J Clin Pathol 1998;110:70–77. 9661924 10.1093/ajcp/110.1.70

[79] Carmeliet E. Cardiac ionic currents and acute ischemia: from channels to arrhythmias. Physiol Rev 1999;79:917–1017. 10390520 10.1152/physrev.1999.79.3.917

[80] Charras G, Paluch E. Blebs lead the way: how to migrate without lamellipodia. Nat Rev Mol Cell Biol 2008;9:730–736. 18628785 10.1038/nrm2453

[81] Charras GT. A short history of blebbing. J Microsc 2008;231:466–478. 18755002 10.1111/j.1365-2818.2008.02059.x

[82] Hickman PE, Potter JM, Aroney C, Koerbin G, Southcott E, Wu AH, Roberts MS. Cardiac troponin may be released by ischemia alone, without necrosis. Clin Chim Acta 2010;411:318–323. 20036224 10.1016/j.cca.2009.12.009

[83] Weil BR, Young RF, Shen X, Suzuki G, Qu J, Malhotra S, Canty JM. Brief myocardial ischemia produces cardiac troponin I release and focal myocyte apoptosis in the absence of pathological infarction in swine. JACC Basic Transl Sci 2017;2:105–114. 28979949 10.1016/j.jacbts.2017.01.006 PMC5624553

[84] Amgalan D, Pekson R, Kitsis RN. Troponin release following brief myocardial ischemia: apoptosis versus necrosis∗. JACC Basic Transl Sci 2017;2:118–121. 29034357 10.1016/j.jacbts.2017.03.008 PMC5637406

[85] Jaffe AS. Another unanswerable question∗. JACC Basic Transl Sci 2017;2:115–117. 10.1016/j.jacbts.2017.03.002 PMC6113556 30167559

[86] McDonough JL, Arrell DK, Van Eyk JE. Troponin I degradation and covalent complex formation accompanies myocardial ischemia/reperfusion injury. Circ Res 1999;84:9–20. 9915770 10.1161/01.res.84.1.9

[87] Mahajan VS, Jarolim P. How to interpret elevated cardiac troponin levels. Circulation 2011;124:2350–2354. 22105197 10.1161/CIRCULATIONAHA.111.023697

[88] Marjot J, Kaier TE, Martin ED, Reji SS, Copeland O, Iqbal M, Goodson B, Hamren S, Harding SE, Marber MS. Quantifying the release of biomarkers of myocardial necrosis from cardiac myocytes and intact myocardium. Clin Chem 2017;63:990–996. 28377413 10.1373/clinchem.2016.264648 PMC6114147

[89] Cheng W, Li B, Kajstura J, Li P, Wolin MS, Sonnenblick EH, Hintze TH, Olivetti G, Anversa P. Stretch-induced programmed myocyte cell death. J Clin Invest 1995;96:2247–2259. 7593611 10.1172/JCI118280 PMC185875

[90] Logeart D, Beyne P, Cusson C, Tokmakova M, Leban M, Guiti C, Bourgoin P, Solal AC. Evidence of cardiac myolysis in severe nonischemic heart failure and the potential role of increased wall strain. Am Heart J 2001;141:247–253. 11174339 10.1067/mhj.2001.111767

[91] Takashio S, Yamamuro M, Izumiya Y, Sugiyama S, Kojima S, Yamamoto E, Tsujita K, Tanaka T, Tayama S, Kaikita K, Hokimoto S, Ogawa H. Coronary microvascular dysfunction and diastolic load correlate with cardiac troponin T release measured by a highly sensitive assay in patients with nonischemic heart failure. J Am Coll Cardiol 2013;62:632–640. 23644085 10.1016/j.jacc.2013.03.065

[92] Feng J, Schaus BJ, Fallavollita JA, Lee TC, Canty JM Jr. Preload induces troponin I degradation independently of myocardial ischemia. Circulation 2001;103:2035–2037. 11319190 10.1161/01.cir.103.16.2035

[93] Hessel MHM, Atsma DE, van der Valk EJM, Bax WH, Schalij MJ, van der Laarse A. Release of cardiac troponin I from viable cardiomyocytes is mediated by integrin stimulation. Pflugers Arch - Eur J Physiol 2008;455:979–986. 17909848 10.1007/s00424-007-0354-8 PMC2226063

[94] Ibanez B, Heusch G, Ovize M, Van de Werf F. Evolving therapies for myocardial ischemia/reperfusion injury. J Am Coll Cardiol 2015;65:1454–1471. 25857912 10.1016/j.jacc.2015.02.032

[95] Wu AHB, Jaffe AS, Apple FS, Jesse RL, Francis GL, Morrow DA, Newby LK, Ravkilde J, Tang WHW, Christenson RH, Cannon CP, Storrow AB. National academy of clinical biochemistry laboratory medicine practice guidelines: Use of cardiac troponin and B-type natriuretic peptide or N-terminal proB-type natriuretic peptide for etiologies other than acute coronary syndromes and heart failure. Clin Chem 2007;53:2086–2096. 17954494 10.1373/clinchem.2007.095679

[96] Khan NA, Hemmelgarn BR, Tonelli M, Thompson CR, Levin A. Prognostic value of troponin T and I among asymptomatic patients with end-stage renal disease: a meta-analysis. Circulation 2005;112:3088–3096. 16286604 10.1161/CIRCULATIONAHA.105.560128

[97] Collinson PO, Gaze DC. Cardiac troponins in patients with renal failure: what are we measuring and when should we measure it? Ann Clin Biochem 2009;46:269–270. 19454536 10.1258/acb.2009.009058

[98] Schiffrin EL, Lipman ML, Mann JFE. Chronic kidney disease: effects on the cardiovascular system. Circulation 2007;116:85–97. 17606856 10.1161/CIRCULATIONAHA.106.678342

[99] Freda BJ, Tang WHW, Van Lente F, Peacock WF, Francis GS. Cardiac troponins in renal insufficiency: review and clinical implications. J Am Coll Cardiol 2002;40:2065–2071. 12505215 10.1016/s0735-1097(02)02608-6

[100] Sharma R, Gaze DC, Pellerin D, Mehta RL, Gregson H, Streather CP, Collinson PO, Brecker SJD. Cardiac structural and functional abnormalities in end stage renal disease patients with elevated cardiac troponin T. Heart 2005;92:804–809. 16216854 10.1136/hrt.2005.069666 PMC1860676

[101] Apple FS, Murakami MM, Pearce LA, Herzog CA. Predictive value of cardiac troponin I and T for subsequent death in end-stage renal disease. Circulation 2002;106:2941–2945. 12460876 10.1161/01.cir.0000041254.30637.34

[102] Gaze DC, Collinson PO. Cardiac troponin I but not cardiac troponin T adheres to polysulfone dialyser membranes in an in vitro haemodialysis model: explanation for lower serum cTnI concentrations following dialysis. Open Heart 2014;1:e000108. 25332816 10.1136/openhrt-2014-000108 PMC4195923

[103] Hochholzer W, Morrow DA, Giugliano RP. Novel biomarkers in cardiovascular disease: update 2010. Am Heart J 2010;160:583–594. 20934551 10.1016/j.ahj.2010.06.010

